# In vitro effects of aqueous extract from *Maytenus senegalensis (Lam.)* Exell stem bark on egg hatching, larval migration and adult worms of *Haemonchus contortus*

**DOI:** 10.1186/s12917-018-1475-3

**Published:** 2018-05-02

**Authors:** Calvin Bogning Zangueu, Abiodoun Pascal Olounlade, Marlyse Ossokomack, Yolande Noelle Nangue Djouatsa, Goue Géorcelin Alowanou, Anatole Guy Blaise Azebaze, Eulogio José Llorent-Martínez, Maria Luisa Fernández de Córdova, Alain Bertrand Dongmo, Mawulé Sylvie Hounzangbe-Adote

**Affiliations:** 1 Laboratory of Animal Biology, Faculty of Science, University of Douala, P.O. Box 24157, Douala, Cameroon; 2 Laboratory of Ethnopharmacology and Animal Health, Faculty of Agronomic Science University of Abomey Calavi, 01 P.O. Box 526, Cotonou, Benin; 3 Pluridisciplinairy Laboratory, School of Management and Exploitation of livestock Systems, University National of Agricultural of Porto-Novo, 01 BP: 55 Porto – Novo, Benin; 4 Laboratory of chemistry, Faculty of Science, University of Douala, Cameroon, P.O. Box 24157, Douala, Cameroon; 5 Department of Physical and Analytical Chemistry, University of Jaén, Campus Las Lagunillas S/N, E-23071 Jaén, Spain

**Keywords:** *Maytenus senegalensis*, In vitro, *Haemonchus contortus*, Anthelmintic activity, Parasitic nematodes

## Abstract

**Background:**

*Maytenus senegalensis* is a common shrub which is scattered in tropical Africa. Different parts of this plant have been reported to be useful in traditional medicine against gastrointestinal disorders and intestinal worms. This study evaluated the anthelmintic activity of the aqueous stem bark extract of *M. senegalensis* using egg hatch assay (EHA), larval migration inhibition assay (LMIA) and adult worms’ motility inhibition assay (AMIA).

**Results:**

On EHA, the extract concentrations tested resulted in a significant (*p* < 0.01) inhibition of egg hatching in concentration-dependent manner and ranged between 31.86% at 75 μg.mL^− 1^ to 54.92% at 2400 μg.mL^− 1^ after a 48 h post-exposure with eggs. For the LMI assays, the aqueous extract of *M. senegalensis* showed a significant (*p* < 0.05) inhibition of larval migration in a concentration-dependent manner. The highest concentration used (2400 μg.mL^− 1^) showed a 37.77% inhibition. The use of polyvinyl polypyrrolidone (PVPP) indicated that tannins and flavonoids were partly involved in the effect since the larval migration was inhibited by 15.5%, but other biochemical compounds were also implicated. On AMIA, *M. senegalensis* was associated with a reduced worm motility after a 24 h post exposure compared to phosphate buffered saline as control (*p* < 0.05). By this time 66.66% of the worms’ were found immotile or dead in the wells containing plant extract at 2400 μg.mL^− 1^. The Phytochemical analysis of aqueous extract of *M. senegalensis* by HPLC-ESI-MS^n^ detected the presence of proanthocyanidins (20%) and flavonoids (> 50%).

**Conclusions:**

These in vitro results suggest the presence of some anthelmintic properties in *M. senegalensis* extract, which is traditionally used by small farmers in west and central Africa. These effects may be due to the flavonoids and proanthocyanidins present in the extract and need to be studied under in vivo conditions.

## Background

Helminth infections or helminthiasis are among the most pervasive infections distressing a large proportion of the world’s population. Parasitic nematode infections are among the most common and economically important infectious diseases of grazing livestock, especially of small ruminants in the tropics and subtropics [1]. Helminthiasis has become a concern and contribute to the prevalence of malnutrition, anemia, eosinophilia and pneumonia [2]. The gastrointestinal helminths mainly subsist in the digestive tract, where they cause the loss of appetite and impairment to gastric function. They thus lead to enormous disorders on host physiology particularly in kids or young animals [3]. Epidemiological studies carried out in Northern Cameroon showed that gastrointestinal helminthiasis was one of the common diseases of sheep and goats with high mortality rates, where up to 75% of mortalities were attributed to helminths infections, particularly haemonchosis and monieziosis [4–6].

The most important worm control strategy worldwide has usually been the chemical control of helminths along with improved management approaches. However, the increase of drug resistance and the impact of conventional anthelmintic on the environment lead to look for alternative or rather complementary strategies against gastrointestinal parasites. These strategies include bioactive plants, which are rich in secondary metabolites. These plants seem to represent a promising option as potential natural treatments used in the form of herbal drugs or food supplements. They can also be used as a source for creating allelochemicals, new marketable anthelmintic for affordable and sustainable control of these parasitic infections. *Maytenus senegalensis (Lam.)* Exell is a shrub or small tree, belonging to the family of Celastraceae. This plant is common and scattered in tropical Africa and South Africa, from Senegal to Eritrea, southern Africa and Madagascar. *Maytenus senegalensis* occurs in Guinean and Sudanese savannahs, on any soil [7, 8]. Often in groups of impenetrable bushes, it is a small tree up to 8 m height. It can be recognized by its pale leathery obovate leaves; the bark is scaly, grey with red slash and flowering during the dry season, after the plant comes into leaf [7, 8]. Different parts of the plant have been reported to be useful in traditional medicine against gastrointestinal disorders and helminths infections in humans and livestock [7–11]. Previous study has reported that the ethanol crude extract of *M. senegalensis* leaves did not show significant activity on eggs hatching and larval development *of H. contortus* [12]. In a more recent study, acetone/water extract of *M. senegalensis* leaves showed significant anthelmintic activity using the larval exsheathment inhibition assay (LEIA) [13]. However the effect of this plant on the third larval migration and adult worms’ motility of parasitic nematodes, as well as the mechanisms by which this plant exerts its anti-parasitic activity are still unknown. Moreover all these studies evaluated the effect of the leaf extract instead of stem bark extracts which are used in the present work.

Given this, attempts have been made to further study the anthelmintic potential of this plant. The present study was therefore undertaken to evaluate the in vitro anthelmintic activity of aqueous extracts of *M. senegalensis* stem bark against *H. contortus.*

## Methods

### Plant collection

The stem bark of *M. senegalensis* was collected in June 2014 at Ngaoundere (Adamaoua Region, Cameroon) and a voucher specimen was deposited at the National Herbaruim Yaounde after botanical identification by Dr. Tsobsala. The stem barks were sliced into pieces and sun-dried 4 h/day for 14 days and grounded into powder using a laboratory grinder.

### Plant extraction

Briefly, the decoction was prepared by boiling 100 g of powdered stem bark of *M. senegalensis* in 1 L of distilled water for 30 min. After cooling to room temperature, the solution was filtered, and the resulting filtrate was lyophilized, obtaining 3.3 g of crude residue extract (3.3% *w*/w yield). The extract was stored at 4 °C and used for different phytochemical and biological assays.

### Phytochemical analysis

All reagents and standards were of analytical reagent (AR) grade unless stated otherwise. Catechin, kaempferol, myricetin, quercetin and rutin were purchased from Sigma-Aldrich (St. Louis, MO, USA) and 200 mg.L^− 1^ stock solutions of each standard were prepared in ethanol (HPLC grade; Sigma). LC–MS grade acetonitrile (CH_3_CN, 99%) (LabScan; Dublin, Ireland) and ultrapure water (Milli-Q Waters purification system; Millipore; Milford, MA, USA) were used for the HPLC-MS analyses.

For HPLC analysis, the residue extracts were dissolved in the initial HPLC mobile phase to obtain solutions of 5 mg.mL^− 1^ concentration.

Reverse phase high- performance liquid chromatography (RP-HPLC) was used to analyze the chemical fingerprint of aqueous extracts of *M. senegalensis* stem bark. The HPLC system included a vacuum degasser, an autosampler and a binary pump (Agilent Series 1100, Agilent Technologies, Santa Clara, CA, USA). A reversed phase Kinetex core-shell C_18_ analytical column of 100 × 2.1 mm and 2.6 μm particle size (Phenomenex, Torrance, CA, USA) and a C_18_ Security Guard Ultra cartridge (Phenomenex) of 2.1 mm i.d. placed before the analytical column were used. The mobile phase consisted of a mixture of water-formic acid (100:0.1, *v*/v) and acetonitrile (ACN). The following gradient program was used: 10% ACN (0 min), 25% ACN (10–20 min), 50% A (40 min), 100% ACN (42–47 min), and back to the initial mobile phase (− 6 min stabilization). The flow rate was 0.4 mL min^− 1^. After filtration through 0.45 μm PTFE membrane filters, 10 μL of each extract was injected.

The HPLC system was connected to an ion trap mass spectrometer (Esquire 6000, Bruker Daltonics, Billerica, MA, USA) equipped with an electrospray (ESI) interface. The scan range was set at m/z 100–1200 with a speed of 13,000 Da/s. The ESI conditions were as follows: drying gas (N_2_) flow rate and temperature, 10 mL/min and 365 °C; nebulizer gas (N_2_) pressure, 50 psi; capillary voltage, 4500 V; capillary exit voltage, − 117.3 V. The auto MS^n^ mode (negative and positive ionization modes) was used for the acquisition, with an isolation width of 4.0 m/z, and fragmentation amplitude of 0.6 V (MS^n^ up to MS^4^). The analysis of the phenolic composition was carried out by HPLC-ESI-MS^n^ using negative and The flow rate was 0.4 mL min^− 1^. The initial step for the characterization of the phenolic compounds consisted in the determination of the molecular weight of each compound. In the negative ionization mode (ESI^−^) MS^1^ spectrum, the most intense peak corresponded to the deprotonated molecular ion [M-H]^−^ or formate adduct [M + HCOOH-H]^−^. Esquire control software was used for the data acquisition and Data Analysis for processing.

### In vitro anthelmintic assays

The anthelmintic activity of the aqueous extract of *M. senegalensis* stem bark was tested on the different life-cycle stages of *Haemonchus contortus* (Rudolphi, 1803) local isolate. The sheep or lambs were acquired from Faculty of Agronomic Science farm located at the main campus of the University of Abomey Calavi, Cotonou-Benin. A complete state of unconsciousness was obtained by using a captive bolt pistol which delivers a force (concussion) into the head of the sheep. The process is known as humane slaughtering. This state of unconsciousness is rendered prior the bleeding through the section of carotid to kill the animal. Adult worms of *H. contortus* were collected from the abomasums of the local sheep. Immediately after slaughtering, the abomasums were collected and transported to the laboratory. Adult female parasites were then selected, washed, and crushed to liberate the eggs. The eggs were then cultured in a glass jar filled with charcoal and wood sawdust powder for 10 days at room temperature. At the end of the 10th day, infective larvae were harvested by sedimentation using Baermann’s devices and kept at 4 °C. About 3000 infective larvae of *H. contortus* (L3) were inoculated to two worm-free djallonké lambs that were kept indoor in separate house in the animal facilities throughout the study. These lambs served as *H. contortus* egg donors and used to fecal culture, infective larvae and adult worms’ collection for subsequent in vitro assays. At the end of this study one of two donor lambs used were humanely killed by semi-decapitation at the slaughterhouse of the farm. The abomasum was removed, the mobile worms were rapidly collected and used on Adult worms Motility Inhibition Assay (AMIA). The one remaining lamb were de-wormed using a single dose of albendazole (5 mg/kg), repeated two weeks later and maintained in quarantine for one month before release in farm. The tests were performed using three different procedures: egg hatch assay (EHA), larval migration inhibition assay (LMIA) and adult worms motility inhibition assay (AMIA).

### Egg hatch assay (EHA)

The eggs used in the present assay were collected from the previously mentioned donor lambs according to the World Association for the Advancement of Veterinary Parasitology (WAAVP) guidelines [14]. The test was performed according to the procedure described by Coles [13]. Eggs suspension was adjusted to 1000 eggs per mL and distributed in 24-multiwell plates (1 mL per well). For the treatment, 1 mL of the aqueous extract prepared with phosphate buffered saline (PBS) at different concentrations (75, 150, 300, 600, 1200, and 2400 μg.mL^− 1^) was added. PBS was used as negative control and thiabendazole (125, 250 and 500 μg.mL^− 1^ in PBS) as positive control. The mixture was then incubated at 27 °C. After 48 h, the egg hatching was stopped by adding two drops of formaldehyde solution (10%) per well. After that, the number of hatched eggs was counted using an optical microscope. The test was repeated five times. The percentage inhibition of hatching (IEO) for each concentration was calculated using the modified formula of Coles [15]: % IEO = 100 (1- X1 / X2), where X1 was the number of eggs hatches in contact with the extracts and X2 the number of eggs hatches with the negative control.

### Larval migration inhibition assay (LMIA)

Infective larvae of *H. contortus* (L_3_) were obtained by fecal culture collected from an experimentally infected lamb at room temperature as described by Kerboeuf [16]. After egg hatching, infective stage was reached after 10 days. The L_3_ were then collected by sedimentation using Baermann’s devices.

The larval migration inhibition assay was performed as described by Rabel [17], adapted for plant extracts [18]. This test is based on the measurement of the rate of migration of larvae through a membrane after contact with the plant extracts. A known quantity of L_3_ larvae (1000/mL) was brought into contact with aqueous extracts at different concentrations (1200, 600, 300, 150 and 75 μg.mL^− 1^) and incubated for three hours at 20 °C. The assay was replicated three times for each extract concentration and the controls. Then, the larval suspension was washed and centrifuged three times with PBS buffer. Each suspension was allowed to migrate through a 20 μm diameter mesh for three hours at 23 °C. PBS and levamisole (250 μg.mL^− 1^) were used as negative and positive control, respectively. After 3 h of incubation, the inserts were removed and the larvae that migrated were included in a volume adjusted to 1.5 mL by adding PBS. After counting the larvae under a magnifying glass, the percentage of larval migration inhibition (LMI) was calculated using the following formula: % LMI = [(T – M) / T × 100], where T is the total number of larvae L_3_ that were in contact with PBS and M is the number of larvae L_3_ in contact with the extracts or levamisole.

### Involvement of the tannins in the anthelmintic activity

Polyvinyl polypyrrolidone (PVPP) forms complexes with tannins and polyphenols and thus blocks their potential biological activity [19]. PVPP was added to the aqueous extract at concentrations of 1200 and 600 μg.mL^− 1^ and kept overnight in a 1:50 ratio [20]. These solutions were then centrifuged (4500 RPM, 5 min, 20 °C), and the supernatant was incubated with infective larvae (L_3_). After that, the LMIA was performed as previously described.

### Adult worms’ motility inhibition assay (AMIA)

After slaughter of experimentally infected lamb, the abomasum was removed, opened and the contents placed in 0.9% saline solution at 37 °C. The mobile worms were rapidly collected, washed, recovered and placed in the saline solution at 27 °C.

The anthelmintic effect of the aqueous extract of plant on adult worm motility was evaluated according to Hounzangbe-Adote [21]. Solutions of the aqueous extract were prepared with PBS at six different concentrations (75, 150, 300, 600, 1200, and 2400 μg.mL^− 1^), and 1 mL of each of these solutions was deposited in titration plate wells. Actively moving adult worms were then placed into each well (one worm/well). PBS and levamisole (125, 250 and 500 μg.mL^− 1^ in PBS) solutions were also prepared and used as negative and positive controls, respectively. The test was repeated six times for each concentration and for controls. The inhibition of motility of adult worms was used as the criterion for anthelmintic activity. After exposing worms to the aqueous extract, motility was observed every 6 h using a magnifying glass. Adult worms’ motility inhibition was evaluated as the following ratio: number of immotile worms divided by the total number of worms for each concentration or control. The death of the worms was determined by the absence of motility for five seconds. The observations ended when all the worms in PBS died.

### Data analysis

The results were summarized as means ± standard error of means, while differences between means were analyzed at the 5% level of significance using the one way analysis of variance (ANOVA) followed by Dunnett’s or Newman-Keuls post hoc test on Graph Pad Prism Version 5.03 software. The concentration of the extract or standard required to inhibit 50% of eggs hatching, larval migration or adult worms’ motility (IC_50_) as well as their 95% confidence intervals (CI) was generated by the logarithmic non linear regression function on Graph Pad Prism Version 5.03 software.

## Results and discussion

**Fig. 1:**
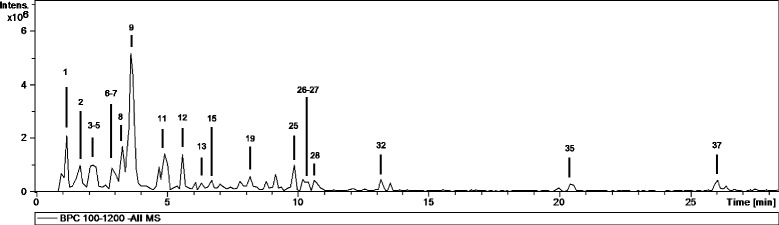
HPLC-ESI/MS^n^ base peak chromatograms (BPC) of the aqueous extract of *Maytenus senegalensis.* In the negative ionization mode (ESI^−^) MS^1^ spectrum, the most intense peak corresponded to the deprotonated molecular ion [M-H]^−^ or formate adduct [M + HCOOH-H]

The qualitative analysis of the aqueous extract of *M. senegalensis* by HPLC-ESI-MS^n^ shown in Table 1 corresponds to the ESI^−^ mode, whereas the ESI^+^ mode was used to confirm the proanthocyanidins structures. Approximately 70 % of the compounds were proanthocyanidins (20%) and flavonoids (> 50%). The base peak chromatogram of the aqueous extract is shown in Fig. 1.

**Table 1:** Chemical characterization of the aqueous extract of *Maytenus senegalensis* stems bark

No.	t_*R*_ (min)	[M-H]^−^ *m/z*	m/z (% base peak)	Assigned identification
1	1.2	191	MS^2^ [191]: 173 (31), 129 (3), 111 (100)	Citric acid
2	1.6	879	MS^2^ [879]: 879 (100), 861 (79), 727 (61), 709 (47), 529 (16), 287 (38) MS^3^ [879 → 861]: 843 (100), 709 (49), 573 (84), 411 (75), 285 (48)	(Epi)gallocatechin-(epi)catechin-(epi)catechin
3	2.3	593	MS^2^ [593]: 575 (29), 467 (31), 441 (63), 423 (100), 305 (64), 289 (7), 287 (7)	(Epi)catechin-(epi)gallocatechin (B-type)
4	2.3	577	MS^2^ [577]: 575 (16), 451 (13), 425 (100), 407 (72), 289 (28), 287 (14), 245 (6)	(Epi)catechin-(epi)catechin (B-type)
5	2.6	865	MS^2^ [865]: 739 (87), 713 (54), 695 (100), 577 (80), 575 (51), 289 (6), 287 (27)	(Epi)catechin-(epi)catechin-(epi)catechin (B-type)
6	3.1	577	MS^2^ [577]: 575 (6), 451 (14), 425 (100), 407 (74), 289 (22), 287 (41)	(Epi)catechin-(epi)catechin (B-type)
7	3.1	289	MS^2^ [289]: 245 (100), 205 (23), 203 (23), 125 (13)	Catechin
8	3.4	595	MS^2^ [595]: 577 (9), 505 (12) 475 (44), 415 (16), 385 (89), 355 (100), 313 (7)	Naringenin-6,8-di-*C*-hexoside
9	3.6	577	MS^2^ [577]: 575 (4), 451 (12), 425 (100), 407 (57), 289 (16), 287 (11)	(Epi)catechin-(epi)catechin (B-type)
10	4.3	581	MS^2^ [581]: 419 (100) MS^3^ [581 → 419]: 373 (14), 404 (100) MS^4^ [581 → 419 → 404]: 389 (100), 373 (38)	Methylated flavonoid-*O*-hexoside
11	4.7	289	MS^2^ [289]: 245 (100), 205 (37), 203 (18), 179 (21), 125 (9)	Epicatechin
12	5.8	865	MS^2^ [865]: 739 (53), 713 (35), 695 (100), 577 (68), 575 (30), 289 (6), 287 (18)	(Epi)catechin-(epi)catechin-(epi)catechin (B-type)
13	6.4	755	MS^2^ [755]: 755 (100), 301 (7), 300 (10) MS^3^ [755 → 300]: 271 (100), 255 (70), 151 (89)	Quercetin derivative
14	6.5	741	MS^2^ [741]: 741 (100), 301 (12) MS^3^ [741 → 301]: 255 (100), 151 (56)	Quercetin derivative
15	6.6	577	MS^2^ [577]: 575 (15), 451 (21), 425 (100), 407 (72), 289 (41), 287 (13), 245 (12)	(Epi)catechin-(epi)catechin (B-type)
16	7.5	739	MS^2^ [739]: 739 (100), 285 (11), 284 (9) MS^3^ [739 → 284]: 255 (100), 241 (17), 151 (28)	Kaempferol derivative
17	7.9	593	MS^2^ [593]: 447 (100), 301 (49) MS^3^ [593 → 447]: 301 (100), 300 (65), 271 (50), 255 (31), 179 (42)	Quercetin-*O*-di-rhamnoside
18	8.0	609	MS^2^ [609]: 301 (100) MS^3^ [609 → 301]: 271 (15), 255 (12), 179 (54), 151 (100)	Rutin
19	8.4	463	MS^2^ [463]: 317 (70), 316 (100) MS^3^ [463 → 316]: 271 (100), 179 (48), 151 (14)	Myricetin-*O*-rhamnoside
20	8.5	593	MS^2^ [593]: 285 (80), 284 (100) MS^3^ [593 → 284]: 255 (100), 151 (23)	Kaempferol-*O*-rutinoside
21	8.8	309	MS^2^ [309]: 193 (100) MS^3^ [309 → 193]: 178 (100), 149 (50), 134 (85)	Ferulic acid derivative
22	9.0	597	MS^2^ [597]: 477 (64), 459 (15), 417 (13), 387 (45), 357 (100), 315 (11)	Phloretin-di-*C*-hexoside
23	9.7	433	MS^2^ [433]: 301 (100), 300 (77) MS^3^ [433 → 301]: 271 (100), 255 (94), 151 (54)	Quercetin-*O*-pentoside
24	9.8	505	MS^2^ [505]: 301 (100), 300 (40) MS^3^ [505 → 301]: 271 (96), 255 (18), 179 (100), 151 (87)	Quercetin-O-acetylhexoside
25	9.9	593	MS^2^ [593]: 447 (100) MS^3^ [593 → 447]: 301 (100), 300 (21) MS^4^ [593 → 447 → 301]: 179 (100), 151 (62)	Quercetin-*O*-di-rhamnoside
26	10.4	563	MS^2^ [563]: 417 (13), 285 (90), 284 (100) MS^3^ [563 → 284]: 257 (74), 255 (100), 227 (84), 151 (41)	Kaempferol-*O*-rhamnosylpentoside
27	10.5	447	MS^2^ [447]: 301 (100) MS^3^ [447 → 301]: 255 (100), 179 (18)	Quercetin-*O*-rhamnoside
28	10.8	577	MS^2^ [577]: 577 (100), 431 (16), 285 (11) MS^3^ [577 → 431]: 284 (90), 285 (100) MS^4^ [577 → 431 → 284]: 255 (40), 151 (100)	Kaempferol-*O*-di-rhamnoside
29	11.0	489	MS^2^ [489]: 285 (66), 284 (100) MS^3^ [489 → 284]: 255 (100), 227 (29)	Kaempferol-*O*-acetylhexoside
30	12.1	431	MS^2^ [431]: 284 (35), 285 (100), 255 (11), 227 (5)	Kaempferol-*O*-rhamnoside
31	12.8	329	MS^2^ [329]: 314 (100) MS^3^ [329 → 314]: 299 (100), 269 (71)	Dimethylated flavonoid
32	13.2	357	MS^2^ [357]:168 (7), 167 (100) MS^3^ [357 → 167]: 123 (100)	Vanillic acid derivative
33	13.6	483	MS^2^ [483]: 437 (100) MS^3^ [483 → 437]: 291 (100)	Flavonoid rhamnoside (formate adduct)
34	19.9	327	MS^2^ [327]: 291 (12), 229 (12), 211 (5), 171 (100)	Oxo-dihydroxy-octadecenoic acid
35	20.5	327	MS^2^ [327]: 291 (24), 229 (79), 211 (47), 209 (10), 171 (100)	Oxo-dihydroxy-octadecenoic acid
36	25.6	329	MS^2^ [329]: 311 (21), 293 (28), 229 (100), 211 (62), 171 (82)	Trihydroxy-octadecenoic acid
37	26.2	397	MS^2^ [397]: 351 (100), 329 (77), 193 (20) MS^3^ [397 → 351]: 251 (87), 233 (78), 193 (100)	Unknown (formate adduct)

The initial step for the characterization of the phenolic compounds consisted in the determination of the molecular weight of each compound. The qualitative analysis of the aqueous extract of *M. senegalensis* by HPLC-ESI-MSn obtained correspond to the ESI^−^ mode, whereas the ESI^+^ mode was used to confirm the proanthocyanidins structures. Approximately 70 % of the compounds detected in the extracts were proanthocyanidins (20%) and flavonoids (> 50%). Esquire control software was used for the data acquisition and data analysis for processing

Compounds **7** and **11** exhibited the deprotonated molecular ion at m/z 289 and identical fragmentation patterns, corresponding to catechin or epicatechin, which cannot be distinguished by their mass spectra. However, considering the elution order [22] and the analysis of a catechin standard they were identified as catechin and epicatechin, respectively.

Compound **8**, with [M-H]^−^ at m/z 595, exhibited MS^2^ fragment ions at m/z 577 [M-H-18]^−^, 505 [M-H-90]^−^, 475 [M-H-120]^−^, 385 [aglycone+ 113]^−^, and 355 [aglycone+ 83]-, typical of di-C glycosyl trihydroxyflavanones or trihydroxyflavones. Taking into account bibliographic information, this compound was tentatively characterized as naringenin-6,8-di-C-hexoside [23, 24].

Eight quercetin derivatives (aglycone at m/z 301), identified by comparison with an analytical standard were observed in the extracts of *M. senegalensis*. Compounds **17** and **25** exhibited the deprotonated molecular ion at m/z 593, and suffered two consecutive losses of 146 Da (593 → 447, 447 → 301), which corresponded to rhamnoside units, so they were characterized as quercetin-*O*-di-rhamnoside isomers. Compounds **23**, **24** and **27** suffered neutral losses of 132, 204 and 146 Da, respectively, to yield quercetin at m/z 301; hence, they were identified as quercetin-*O*-pentoside, quercetin-*O*-acetylhexoside and quercetin-*O*-rhamnoside, respectively. The structures of compounds **13** and **14** could not be fully elucidated. Compound **18** was identified as rutin by comparison with an analytical standard.

Six kaempferol derivatives (aglycone at m/z 285, comparison with an analytical standard) were detected. Compound **16** suffered the neutral loss of 454 Da, probably rhamnoside-hexoside-rhamnoside, but without further information it was merely characterized as a kaempferol derivative. Compounds **20**, **29** and **30** suffered neutral losses of 308 Da, 204 Da and 146 Da, respectively; hence, they were characterized as kaempferol-*O*-rutinoside, kaempferol-*O*-acetylheoside and kaempferol-*O*-rhamnoside, respectively. Compound **26** exhibited MS^2^ neutral losses of 146 Da (563 → 417; rhamnoside) and 278 Da (563 → 285; rhamnoside+pentoside), so it was tentatively characterized as kaempferol-*O*-rhamnosylpentoside. Finally, compound **28** exhibited two consecutive neutral losses of 146 Da, so it was identified as kaempferol-*O*-di-rhamnoside.

Compound **19** exhibited the deprotonated molecular ion at m/z 463 and suffered the loss of a rhamnoside unit in MS^2^, yielding the aglycone at m/z 317, which corresponded to myricetin (fragment ions at m/z 271, 179 and 151; comparison with an analytical standard), so it was characterized as myricetin-*O*-rhamnoside.

Compound **21** exhibited its base peak at m/z 193, which displayed MS^3^[309 → 193] fragment ions at m/z 178, 149 and 134, typical of ferulic acid, so it was characterized as a derivative.

Compound **22**, with [M-H]^−^ at m/z 597, presented MS^2^ fragment ions at m/z 477 [M-H-120]^−^, 417 [M-H-180]^−^, 387 [aglycone+ 113]^−^ and 357 [aglycone+ 83]^−^, typical of di-*C*-glycosides. Considering bibliographic data [25], it was tentatively characterized as phloretin-di-*C*-hexoside.

Six proanthocyanidins dimers and two trimers, all of them B-type, were characterized in the extracts. Compound **2**, with [M-H]^−^ at m/z 879, was tentatively characterized as a trimer of the (epi)gallocatechin-(epi)catechin-(epi)catechin type after comparison with bibliographic data [26]. Compounds **4**, **6**, **9**, and **15** exhibited their base peak at m/z 577, and fragment ions at m/z 451, 425, 407, and 289, characteristic of (epi)catechin-(epi)catechin procyanidin dimers [27, 28]. Compound **3** was identified as (epi)catechin-(epi)gallocatechin considering its [M-H]^−^ ion at m/z 593 and its fragmentation pattern [29], in which neutral losses of a galloyl group (152 Da) and an (epi)gallocatechin unit (304 Da) were observed. Compounds **5** and **12** displayed their [M-H]^−^ at m/z 865, and exhibited two consecutive losses of (epi)catechin units; they were characterized as procyanidin trimers (epi)catechin-(epi)catechin-(epi)catechin [27, 28].

Besides phenolic acids, flavonoids and proanthocyanidins, other compounds were also found. Compound **1** was identified as citric acid due the deprotonated molecular ion at m/z 191 and its characteristic MS^2^ base peak at m/z 111 [30].

Compound **32** exhibited the deprotonated molecular ion at m/z 357, and was tentatively characterized as a vanillic acid derivative due to the 167 → 123 transition observed in its MS^n^ fragmentation.

Three oxylipins were detected in the samples, and characterized by comparison of their mass spectra with bibliographic data [31]. Compounds **34** and **35** were characterized as oxo-dihydroxy-octadecenoic acids, whereas compound **36** was identified as trihydroxy-octadecenoic acid.

**Fig. 2:**
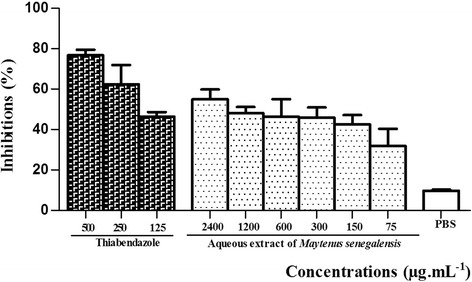
Inhibition of egg hatching with various concentrations of the aqueous extract of *Maytenus senegalensis* and thiabendazole. Compared to PBS control, the inhibitory effects were significant (*p* < 0.01) for thiabendazole and the aqueous extract of *Maytenus senegalensis*. Each bar represent mean ± SEM; p < 0.01 when compared with PBS follow by Dunnett post test; SEM = standard error of mean; PBS = phosphate buffered saline

The extract of *M. senegalensis* containing all these compounds was used to evaluate his anthelmintic effect as claimed by traditional healers or farmers in different parts of Africa. For in vitro studies, *H. contortus* proved to be a good test worm because of its longer survival in PBS. This worm and some other *Strongyloides* have previously been used for in vitro studies [32, 33]. The different trials were performed to screen the capacity of the aqueous extract of *M. senegalensis* stem bark to disrupt the life cycle of *H. contortus*. Concentration of the aqueous extract of *M. senegalensis* between 75 to 2400 μg.mL^− 1^ showed significant anthelmintic activity on eggs hatching, larval migration and adult worms’ motility assay. Forty eight (48) hours post exposure of eggs with plant extract resulted in an ovicidal effect as consequence of the inhibition of eggs hatching. The plant extracts showed significant (*p* < 0.01), concentration-dependent egg hatching inhibition at the tested concentrations (Fig. 2). This effect ranged between 31.86% at 75 μg.mL^− 1^ and 54.92% at 2400 μg.mL^− 1^ of plant extract. In the negative control (PBS), the mean hatching inhibition rate of the eggs was estimated at less than 10%. The effect shown by the extract upon egg hatch in the present study seems to contradict an earlier work that evaluated the ovicidal activity of the ethanol extract of this plant (leaves) on *H. contortus* [12]. A possible explanation could be the high concentrations of *M. senegalensis* extract (75 to 2400 μg.mL^− 1^) used in the present study compared to the concentration of extract (1.3 to 1700 μg.mL^− 1^) used in the previous study*.* In the present study, the concentration of the extract required to inhibit 50% of eggs (IC_50_) from hatching known as lethal concentration 50 (LC_50_) was 60.75 μg.mL^− 1^. Another fact that deserves to be emphasized is the nature of the extract used; the active principles of the aqueous extract (used in this study) could be different to those found in the ethanol extract in terms of quality and quantity.

Thus, the stem bark of *M. senegalensis*, used as herbal drug or combined to food supplements, can help to modulate helminthiasis by the use of long term treatments of animals in a given farm. These treatments may reduce hatchability of the eggs excreted in the faeces, resulting in both a reduced risk of re-infection and lighter worm loads by decreasing pasture contamination.

**Fig. 3:**
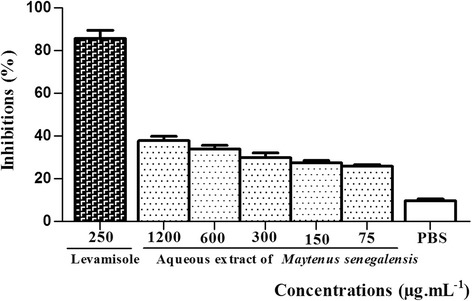
Inhibition of larval migration with various concentrations of the aqueous extract of *Maytenus senegalensis* and Levamisole. Significant effects were observed for levamisole (p < 0.01) and aqueous extract of *Maytenus senegalensis* (*p* < 0.05) when compared to control (PBS). Each bar represents mean ± SEM; *P* < 0.05 when compared with PBS follows by Dunnett post test. SEM = standard error of mean; PBS = phosphate buffered saline

**Fig. 4:**
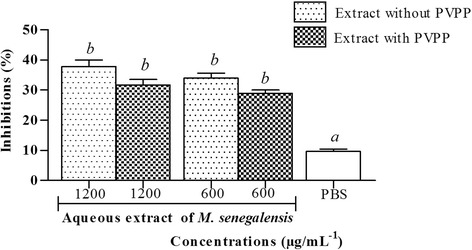
Inhibition of larval migration with the aqueous extract of *Maytenus senegalensis* without and with polyvinyl polypyrrolidone. Each bar represents mean ± SEM. No significant effects were observed for aqueous extract of *Maytenus senegalensis* pre-treated with polyvinyl polypyrrolidone (PVPP) (*p* > 0.05), when compared to aqueous extract without PVPP using Newman-Keuls post test. PBS was used as negative control. Different superscripts indicate significant differences (*p* < 0. 05) between treatments followed by Dunnett’s post test. *M. senegalensis = Maytenus senegalensis*. SEM = standard error of mean; PBS = phosphate buffered saline

The effects of the plant aqueous extract on third - stage larvae were evaluated by the LMI test. The aqueous extract of *M. senegalensis* (at all the tested concentrations), significantly disrupted larval migration of L_3_ through 20 μm diameter mesh sieves (Fig. 3). PBS did not affect significantly larval migration (less than 10%), while aqueous extract of *M. senegalensis* presented a significant (*p* < 0.05) concentration-dependent effect, with inhibition from 25.80 to 37.77%, as compared to control. Levamisole, used as positive control, exhibited a more significant effect (*p* < 0.01) with 85.55% larval migration inhibition. The concentration of the extract or thiabendazole required to inhibit 50% of eggs (IC_50_) from hatching, was calculated as 18.57 and 60.75 μg.mL^− 1^, respectively (Table 2). The decrease of larval migration induced by the plant extract could be associated with the ability of its bioactive compounds to exhibit a larvicidal activity as a consequence of larval mortality or larval paralysis. The activity shown by the extract in the present study agrees with an earlier work that reported a significant larvicidal effect of acetone/water extracts of the leaves of this plant on infective larvae of *H. contortus* using LEIA [13]. The aqueous extract of *M. senegalensis* showed a larvicidal effect with and IC_50_ value of 70.79 μg.mL^− 1^ (Table 2), and appears to be modest compared to the results reported by the previous study [13]. The importance of the effect observed could be due to the nature of the test used, since the mechanism involved in the larval exsheathment assay is not exactly the same as that of the larval migration assay. Moreover, the various parts of the plant (leaves vs stem bark) and the type of solvent (acetone-water vs water) used for the extraction was different. Thus, the secondary metabolites present in the plant extract (regarding both quality and quantity) could differ from one extract to another. Water was used as the extractant in the current study, in the same way that it is applied by local communities. The ability of this solvent to extract compounds of a wide polarity range at a high yield is limited compared to acetone or acetone-water used in similar studies [13, 34, 35]. Acetone was selected by these authors as a suitable extractant due to its ability to extract compounds of a wide polarity range, a better solvent for plant secondary compounds than water, which is the common solvent used by rural communities. In order to specify the contribution of some bioactive compounds associated to larval migration inhibition or larvicidal activity, PVPP was added to the extract and LMIA was performed. PVPP forms complexes with tannins and polyphenols and thus blocks their potential biological activity [19]. In the presence of PVPP, the effect of extract on larval migration inhibition was reduced from 37.77 to 31.72% and from 33.95 to 28.88% at 1200 and 600 μg.mL^− 1^ concentrations, respectively, when compared to PBS (Fig. 4). After a 30 min post exposure on adult worms’, levamisole (125–500 μg.mL^− 1^) showed an IC_50_ or LC_50_ of 43.79 μg.mL^− 1^. Moreover aqueous extracts of *M. senegalensis* (75–2400 μg.mL^− **1**^**)** showed an IC_50_ or LC_50_ value of 146.2 μg.mL^− **1**^(Table 2). No significant effects were observed for aqueous extract of *M. senegalensis* pre-treated with PVPP (*p* > 0.05). The effect of PVPP on larval migration inhibition showed a 15.5% reduction, when compared to aqueous extracts without PVPP. This partial restoration towards the control values was interpreted as the sign that *M. senegalensis* tannins and/or polyphenols are involved modestly or partially in the anti-parasitic activity observed. The quantity of PVPP used might have been insufficient to inactivate all the tannins and phenolic compounds in the tested extract. Another explanation could be the presence of secondary metabolites, other than tannins and flavonoids, which have potential efficacy against *H. contortus.* Similar results were shown previously by Barrau et al. [20], who found that in *O. viciifolia*, besides tannins and flavan-3-ols, some flavonol glycosides also had an effect on gastrointestinal nematodes. In other similar studies using PVPP to test the involvement of tannins and phenolic compounds in the antiparasitc activity like in the current study, only a partial restoration towards control values was observed after addition of PVPP. This was observed for chestnut or pine tree extracts when using the LEIA [36] or with *Pistacia lentiscus* and *Ceratonia siliqua* with the LMIA [37]. This has led to the conclusion that besides the proanthocyanidins (tannins) and phenolic compounds (flavonoids) present in *M. senegalensis*, other biochemical compounds might also be responsible for the anthelmintic properties since some inhibition of larval migration was still observed. In addition, due to the high diversity of plant secondary compounds in *M. senegalensis*, other components related to the presence of kaempferol, quercetin, myricetin and rutin could also be responsible for part of the anthelmintic activity [20].

As shown in Table 3, the aqueous extract of *M. senegalensis* was associated with a reduced worm motility after 24 h post-exposure compared with the negative control (*p* < 0.05), while 66.66% of worms were found immotile or dead in the wells containing plants extracts at 2400 μg.mL^− 1^. By this time, levamisole exhibited 100% inhibition at 250 μg.mL^− 1^ compared to PBS. The worms were found completely immotile in PBS after 48 h post-exposure. The reduction of motility and the immotility is an indicator of paralysis or mortality of the worms and an evidence of the effect of the plant extract. Nematode muscles are known to contain excitatory neuromuscular junctions showing various receptors with acetylcholine as their neurotransmitter [38]. Some of these metabolites acting as ganglion stimulant could tend to activate these neuromuscular junctions causing a spastic paralysis in the worms leading to their death. Moreover the death could also be due to the flaccid paralysis. Thus, further experiments will be done to elucidate the mechanisms leading to the paralysis of the worms.

**Table 3:** Effects of various concentrations of aqueous extract of *Maytenus senegalensis* on adult worms of *Haemonchus contortus*

Treatments	Concentrations (μg.mL^−1^)	Percentage of immotile worms (%)			
		12 h	24 h	36 h	48 h
PBS	0	0	0	16.66	100
Levamisole	500	66.66	100	100	100
	250	50	100	100	100
*M. senegalensis*	125	33.33	83.33	100	100
	2400	0	66.66	100	100
	1200	16.66	33.33	100	100
	600	0	16.66	83.33	100
	300	0	0	66.66	100
	150	0	0	66.66	100
	75	0	0	50	100

Six replicate per treatment concentrations were used. Data are expressed as a percentage of immotile worms compared to the total number in the wells. PBS = phosphate buffered saline; *p* < 0.05 when compared with PBS follows by Dunnett’s post test

**Table 2:** Extract concentration required to inhibit 50% of effect on various anthelminthic assay (IC_50_) against *Heamonchus contortus* for the *Maytenus senegalensis* extract

Assay	Treatment	IC_50_ (μg.mL^−1^)	CI (95%)		R^2^
			Lower (μg.mL^−1^)	Upper (μg.mL^− 1^)	
EHA	thiabendazole	18.57	10.00	34.48	0.8254
	*M. senegalensis*	60.75	23.93	154.2	0.6258
LMIA	*M. senegalensis*	70.79	37.78	132.7	0.9165
AMIA	levamisole	43.79	23.46	81.76	0.9944
	*M. senegalensis*	146.2	95.01	224.8	0.9684

The concentration of the extract or standard required to inhibit 50% of eggs hatching, larval migration or adult worms’ motility (IC_50_) as well as their 95% confidence intervals (CI) were generated by the logarithmic non linear regression function on Graph Pad Prism Version 5.03 software

The phytochemical analysis of the stem bark of *M. senegalensis* indicated the presence of different metabolites, such as proanthocyanidins and flavonoids. It has been reported that phenolic compounds, including gallotannins, condensed tannins, and flavonoids have been implicated in pharmacological activities such as anthelmintic activities [39]. Tannins are able to bind to the free proteins available in the tube for larval nutrition, and the reduced nutrient availability could lead to larval starvation and death. The ingestion of condensed tannins, which have the capacity to bind to the cuticle, the intestinal mucosa of larvae or adult worms’ which is high in glycoprotein can cause their paralysis or death [40].

On the other hand, the inhibition of egg hatching, larval migration and adult worms’ motility of *H. contortus* could also be associated with esters of some derivative acids or non protein amino acids as previouly reported [41, 42]. The chemical structure of some derivatives of acids (ferulic acid, citric acid and vanilic acid) present in *M. senegalensis* is close to that of the nematicidal compound like kainic acid [41]. These derivatives acids, which can act like kainic acid, could have a neurodegenerative action on nematodes by the substitution of glutamate [41]. In a recent study, esters of gallic acid or gallic acid derivatives have also been associated with anthelmintic properties measured by the inhibition of egg hatching and larval motility of *H. contortus* [43]. This has led to the attest that the synergy of several compounds could contribute to the anthelmintic properties in *M. senegalensis.*

## Conclusions

The use of the aqueous extracts of the stem bark of *M. senegalensis* may therefore be useful for the control of gastrointestinal nematode in livestock production. This anthelmintic activity against egg hatching, infective larvae migration and adult worms’ motility of *H. contortus* is attributed partly to proanthocyanidins and flavonoids (such as glycosides from kaempferol, quercetin, myricetin) as well as to other non-phenolic components that may contribute to exert anthelmintic effect. Further research is needed to characterize and better understand the nature of the secondary metabolites responsible for the anthelmintic effect and to analyse their mode of action on the nematodes.

## Abbreviations

ACN: Acetonitrile
AMIA: Adult worms’ motility inhibition assay
ANOVA: Analysis of variance
AR: Analytical reagent
CI: Confidence intervals
EHA: Egg hatch assay
ESI: Electrospray ionization
*H. contortus*: *Haemonchus contortus*
HPLC: High-performance liquid chromatography
HPLC-ESI-MS: High-performance liquid chromatography-electrospray ionization-mass spectrometry
HPLC-MS: High-performance liquid chromatography-mass spectrometry
LEIA: Larval exsheathment inhibition assay
LMIA: Larval migration inhibition assay
*M. senegalensis*: *Maytenus senegalensis*
PBS: Phosphate Buffered Saline
PVPP: Polyvinyl polypyrrolidone
RP-HPLC: Reverse phase high-performance liquid chromatography
WAAVP: World association for the advancement of veterinary parasitology
